# Sensitivity to changes in dynamic affordances for walking on land and at sea

**DOI:** 10.1371/journal.pone.0221974

**Published:** 2019-10-17

**Authors:** Hannah J. Walter, Nicolette Peterson, Ruixuan Li, Jeffrey B. Wagman, Thomas A. Stoffregen

**Affiliations:** 1 Affordance Perception-Action Laboratory, School of Kinesiology, University of Minnesota, Minneapolis, Minnesota, United States of America; 2 Department of Psychology, Illinois State University, Normal, Illinois, United States of America; Purdue University, UNITED STATES

## Abstract

We investigated the perception of affordances for walking along a narrow path. We asked whether participants could perceive changes in affordances brought about by manipulation of properties of the body, or of the environment, without direct practice of the to-be-perceived affordance, and without external feedback about the accuracy of perception. In Experiment 1, participants made a series of 8 judgments of how far they could walk along a narrow path either, 1) without added weight, 2) while wearing a weighted vest, or 3) while wearing weights on their ankles. Before walking, mean judgments were lower when wearing weights than in the no-weight condition. In addition, in both weight conditions judgments changed across the series of 8 judgments, in the direction of greater accuracy. Control of the body in walking also can be influenced by motion of the ground surface, as commonly happens in vehicles. In Experiment 2, on a ship at sea, we evaluated the effects of walking with or without weight added to the body at the ankles. We again asked participants (experienced maritime crewmembers) to judge how far they could walk along a narrow path, with versus without ankle weights. As in Experiment 1, judgments made before walking mirrored the observed differences in walking performance. In addition, we again found evidence that judgment improved (without walking practice, or feedback) over the series of judgments. We conclude that participants were sensitive to (and spontaneously learned about) how affordances for walking were influenced by changes in the dynamics of body and the environment.

## Introduction

Affordances are possibilities for action that exist for a given animal in a given environment [1–2]. For example, affordances for locomotion emerge from relations between properties of an animal and properties of the environment that allow for movement from place to place [3–4]. One type of locomotion is walking.

Walking includes lateral oscillations of the body as weight shifts between the feet. Walking along a narrow path (for example, a gap between buildings, or along a balance beam) will be constrained by the walker’s ability to control these lateral oscillations, so as to avoid bumping into walls (when walking between buildings) or falling off the path (when walking on the beam). The greater the person’s ability to control lateral oscillations, the farther they can walk along a narrow path.

In the present study, we considered effects of lateral oscillations on affordances for walking a maximum distance along a narrow path [5–6]. We know of no existing research on perception of affordances relating to how far a person on land can walk along a narrow path in a given situation or under given circumstances. There is a considerable empirical literature on step width, but this research typically has addressed clinical issues, in part because control of lateral oscillation rarely is an issue for healthy adults [7–8]. However, environmental conditions can challenge the control of lateral oscillations in walking. An example is walking on a ship at sea.

Humans have been going to sea for many thousands of years [9]. Ocean swells and waves generate oscillatory motion of ships, with motion excursions typically on the order of meters. Ship motion varies with changes in wind, waves, ship speed and heading, among other factors, but with rare exceptions (e.g., a calm) is present around the clock, day in and day out, for the duration of a voyage [10]. This highly complex motion of the deck surface is associated with broad changes in perceptual-motor control. For example, the rolling gait of fully adapted mariners often is visible to casual observers [11]. Recent research on ships at sea has shown that experienced mariners perceive how oscillatory ship motion alters the maximum distance that they can walk along a narrow path. In such studies, independent variables were properties of ship motion [5–6]. In the present study, we asked how affordances for walking along a narrow path might be influenced by changes in properties of the body.

### Added mass and affordances for walking

Changes in the body’s mass (and mass distribution) tend to influence perception and performance of available actions [12]. For example, Regia-Corte and Wagman [13] asked participants to wear a backpack apparatus to which masses were attached in one of three configurations—high-mass, low-mass, or no-mass. In each condition, participants adjusted the angle of inclination of a surface until they felt that it was just barely possible for them to stand on that surface. Perception of affordances for standing on the inclined surface reflected the changes in center of mass brought on by the weighted backpack apparatus—the perceptual boundary occurred at a smaller angle of inclination in the high-mass condition than in either the low-mass condition or the no-mass condition.

### Learning about changed affordances

In Walter et al. [5–6], participants were experienced mariners who were fully adapted to ship motion. The impressive sensitivity of these participants raises questions about how people learn about changes in affordances for walkable distances over both short and long time scales. Mark [14] asked standing participants to judge their maximum sitting height, that is, the highest chair on which they could sit. Participants made a series of judgments. In one condition, participants made these judgments while wearing 10 cm blocks on their feet, which increased actual maximum sitting height. In the initial judgments while wearing the blocks, participants’ responses reflected their sitting ability without the blocks. That is, initial judgments were underestimates. However, across the series of judgment trials, judgments gradually improved. This gradual improvement was remarkable because participants received no feedback about the accuracy of their judgments, and were not permitted to practice sitting while wearing the blocks. Mark et al. [15–17] showed that exploratory movement of the body (e.g., postural sway) was both necessary and sufficient for this gradual retuning of judgments.

We asked whether spontaneous learning of affordances for walking (i.e., without feedback about judgment accuracy) would occur in the context of dynamic changes (in terms of the effect of mass distribution of the body on lateral oscillations) rather than geometric changes (in terms of the effect of between leg length on stepping height). The walking task developed by Walter et al. [5–6] focused on dynamically defined affordances for walking, with independent variables in properties of the environment (aspects of ship motion). In Experiment 1 of the present study, we used a similar walking task, with independent variables in properties of the body; specifically, weights added to the body that were expected to influence walking ability [13, 14–15]. In Experiment 2, we used a similar methodology on a ship at sea: Our aim was to assess sensitivity to the simultaneous influence of dynamic properties of the body (added weight) and dynamic properties of the environment (ship motion).

## Experiment 1

In Experiment 1, our principal purposes were to 1) evaluate effects of added weight on participants’ ability to walk within a narrow path, and 2) to investigate whether actual differences in performance were reflected in prior judgments of walking ability. We predicted that added weight would reduce the distance that participants could walk within the narrow path. We also predicted that effects of the weights would be reflected in judgments of maximum walkable distance.

Mass can be added to the torso [18–21] or to the lower extremities (e.g., heavy ski boots, medical walking boots, or fitness weights on the ankles). We reasoned that, in the context of our path-following task, a greater challenge to lateral gait would arise from weight added to the ankles than to the torso but that there would be greater changes to perceptual information during postural sway from weights added to the torso than to the ankles. For this reason, in Experiment 1 we separately evaluated effects on perception and performance of weights added at the upper torso, and at the ankles.

Our method was based upon prior studies of both perception and performance on land [14–15], and at sea [5–6]. However, we modified the method to take into account the nature of our manipulations in the present study. Given that the addition of weights was sudden and discrete, such that participants had little or no prior exposure to gait while wearing the weights [18, 22], we expected that performance (actual walking ability, in terms of distance walked and the speed of walking) might change across performance trials. In addition, following previous studies [14–15], we expected that the accuracy of judgments might change over the course of a series of judgments. For this reason, we asked participants to make a series of eight judgments before engaging in any actual walking. We predicted that judgments would change, over trials, in conditions with added weight, but that they would not change in the baseline condition (no added weight). Following Mark et al. [15], we evaluated these predictions in terms of the slope of the line across the series of eight judgments for each condition.

In both experiments, to account for our use of a within-participants design, for statistically significant effects we estimated effect size using the *F*-value and its degrees of freedom [23]. Similarly, we computed effect sizes for post-hoc *t*-tests using Cohen’s *d*_*z*_ [23].

## Method

### Participants

**Fig 1:**
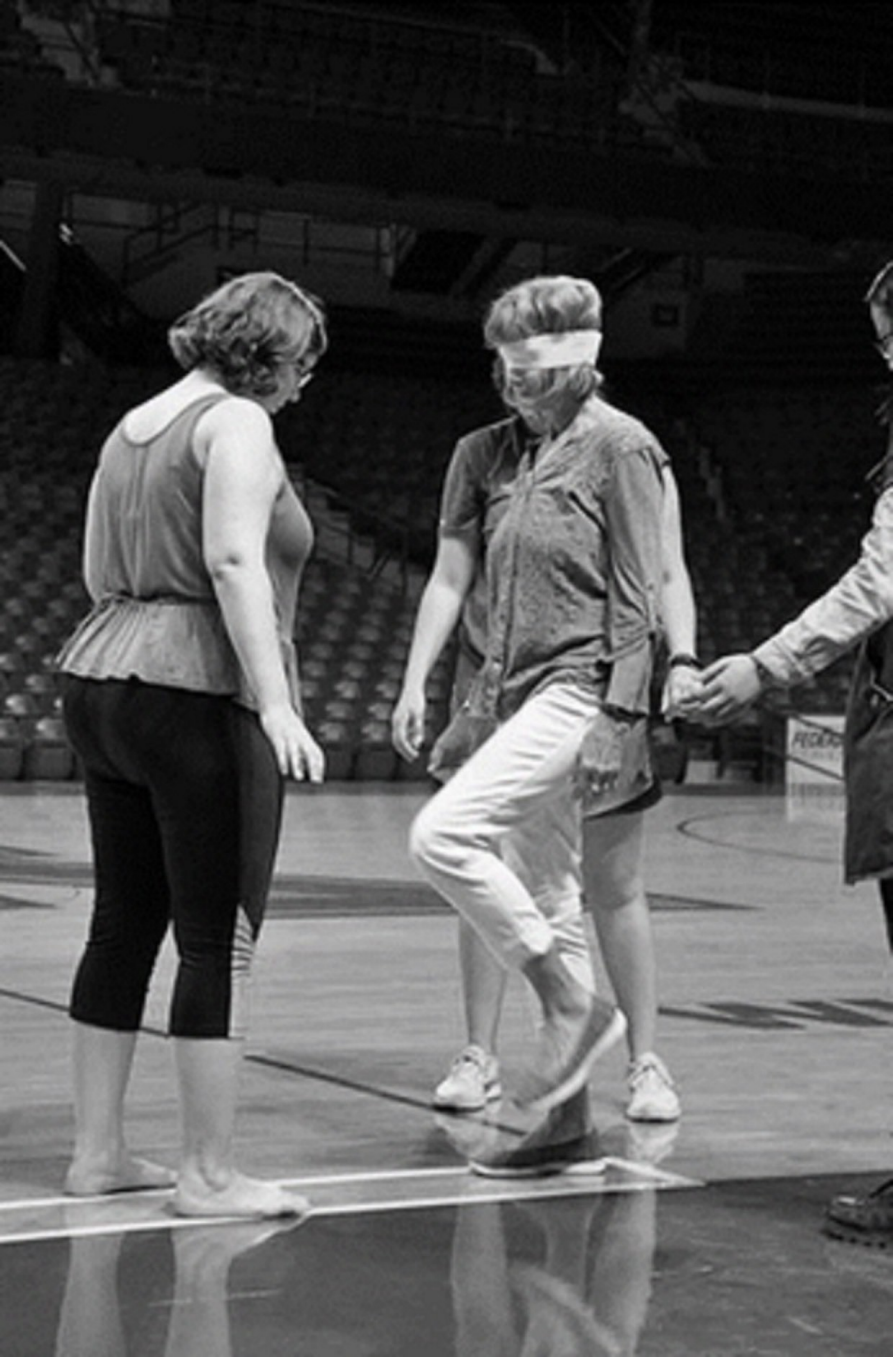
Experiment 1. A participant stepping in place while blindfolded.

Our sample comprised 14 individuals (5 men and 9 women), ranging in age from 18 to 76 years (mean = 39.21 years), in height from 1.44 to 1.81 m (mean = 1.65 m) and in weight from 49.90 kg to 103.41 kg (mean = 70.18 kg). We selected this age range of participants in Experiment 1 to match those of the crewmembers on the ship who would serve as participants in Experiment 2 [5–6, 24]. As part of the consent process, participants indicated that they suffered from no history of balance disorders, vestibular dysfunction, seizures, or dizziness. The experimental protocol was approved in advance by the University of Minnesota IRB. The individual in Fig 1 has given written informed consent (as outlined in PLOS consent form) to publish their image alongside the manuscript.

To ensure a large enough sample size to provide sufficient power reliably to exclude false rejection of the null hypothesis, we tested power (1-β) with the G*Power program [25], using the *a priori* option and the effect size (.81) for affordance judgments from Walter et al. [5] (*n* = 16). Power analysis revealed a test power of .967 and suggested that a sample size of *n* = 14 would be sufficient to achieve the desired effect size of 0.81.

### Setting and apparatus

The study was conducted on an indoor basketball court. In some conditions, athletic weights were applied to the body. We used a weighted vest (j/fit, Vancouver WA), in which 9.1 kg were distributed symmetrically left to right, and front to back. We also used two soft, wrap-around athletic weights (Synergee, Thunder Bay Ont) each 4.55 kg, that could be secured at the ankle using Velcro.

### Procedure

We created a pathway using matte tape on the court, parallel to the sidelines. Following previous studies [5–6], the pathway was 8.9 m long × 0.3 m wide. We used a within-participants design. Participants were tested with their shoes on (at sea, in Experiment 2, this was required). In the No-Weight condition, the participant wore No-Weights attached to the body. In the Torso-Weight condition, the participant wore the weighted vest. In the Ankle-Weight condition, the participant wore one 4.55 kg weight attached at each ankle.

### Stepping in place

To experience how the weights influenced movement in general, participants stepped in place. To ensure that this experience could not provide direct information relating to visible distance, participants did so while blindfolded. The blindfold was removed after stepping, thereby making information about relations between the weights and the (visible) path available during judgments.

Stepping in place was conducted with the participant standing at the beginning at one end of the path, with their heels on a marked line. A blindfold (an opaque elastic head band) was applied, after which (in the Torso-Weight and Ankle-Weight conditions) weights were attached. With the blindfold in place, the participant engaged in stepping in place, that is, sequentially raising each foot off the ground and returning it to its original position (Fig 1). This took place in all conditions, even in the No-Weight condition. Five step cycles were executed (i.e., the right and left feet each were raised 5 times for a total of 10 steps). After stepping in place, the Experimenters assisted the participant in returning their feet to the starting position. Then, the blindfold was removed.

### Judgment task

The participant was asked to look at the designated path and estimate “if you were walking comfortably, how far do you think you could walk along this path without stepping on or over the lines?” To report estimated distance, the participant instructed an experimenter where to place a marker (a 0.25 m length of a wooden 4 × 4) along the path. At the beginning of the trial, the experimenter stood near the participant, facing them, and slowly walked backward along the path until instructed to stop by the participant. Each participant gave eight judgments for each condition (No-Weight, Torso-Weight, Ankle-Weight), for a total of 24 judgments. Across participants, we counterbalanced the order in which the three conditions were presented. We repeated the six possible condition orders in a fixed sequence across successive participants.

### Performance (walking) task

After completing the judgment task, participants were asked to walk comfortably along the path: “Please do not look at your feet. Keep your eyes on the end of the path and walk so as to avoid stepping on the lines.” For each condition, the participant completed a total of 12 trials, comprising six laps (out and back). Stepping on or over the lines with any part of either foot was classified as a “fault” and the walked distance was recorded from the spot of the fault. For each trial, the participant indicated that they were ready, after which the Experimenter gave a “go” signal and started a handheld stopwatch. Each of three experimenters watched for faults, with one experimenter on each side, walking behind so as to be able to monitor footfalls while remaining outside the participant’s field of view, while one experimenter remained at the starting point. The stopwatch was stopped when the participant crossed the end line or when a fault was verbally indicated, and the duration of the trial was recorded.

### Data analysis

Data was deposited in DRUM (Data Repository for University of Minnesota), and can be found online [26]. Our analysis was modeled after that of Mark et al. [15]. Mark et al., did not directly investigate whether their principal manipulation (the wearing of 10 cm blocks on the feet) influenced judgments, relative to a control condition in which the manipulation was absent (the no block condition). In each condition, actual maximum sitting ability did not change from trial to trial. By contrast, in our study actual walking ability could vary from trial to trial [27], especially in Experiment 2 (due to dynamic variation in ship motion) and might also change systematically across actual walking trials (i.e., with practice).

For these reasons, we focused on whether and how the weight manipulations influenced perceived and actual walking ability (with separate ANOVA on means for judgments and for performance) and on whether there were changes across the sequence of judgment trials. Investigating whether the weight manipulations affected actual walking ability (performance), and whether any changes in walking ability were reflected in changes in mean judgments required that we use within-participants design. Consequently, there was an issue of possible order effects in the presentation of the three experimental conditions. Following Mark et al. [15], we evaluated effects of our manipulations in terms of main effects in analysis of variance (ANOVA). For each condition, we calculated means for the eight judgments. We conducted a 3 × 6 ANOVA on these values with factors Condition (No-Weight, Torso-Weight, Ankle-Weight) and Condition Order (1–6). To account for our use of a within-participants design, for statistically significant effects we estimated effect size using the *F*-value and its degrees of freedom [23]. Similarly, we computed effect sizes for post-hoc *t*-tests using Cohen’s *d*_*z*_ [23].

## Results

### Mean judgments

**Fig 2:**
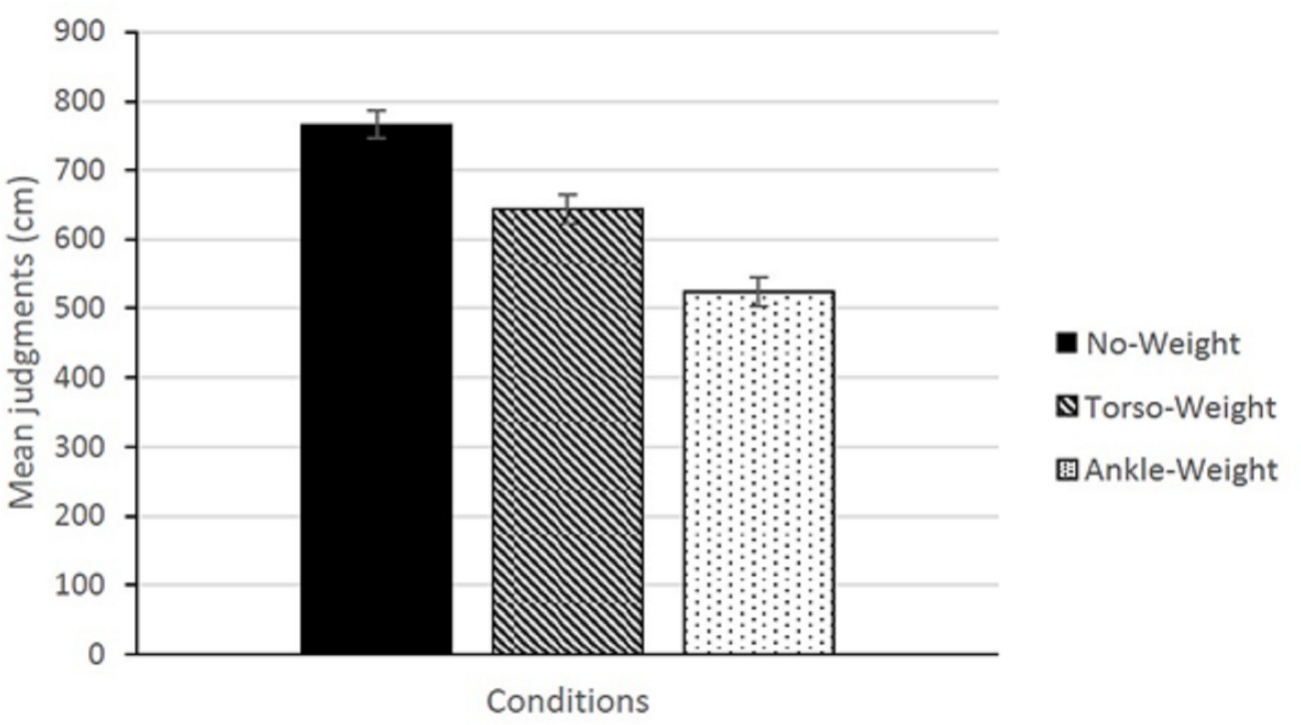
Mean judgments, across participants and judgment trials, of walkable distance. The error bars represent the standard error of the mean.

Collapsed across trials, the judgment data are summarized in Fig 2. The main effect of Conditions was significant, *F*(2,16) = 24.20, *p* < .001, partial η^2^ = 0.75. The main effect of Condition Order, and the Conditions × Condition Order interaction were not significant. None of the participants gave the maximum judgment (890 cm) for each judgment in every condition; that is, no participant exhibited a ceiling effect.

### Changes across judgment trials

**Fig 3:**
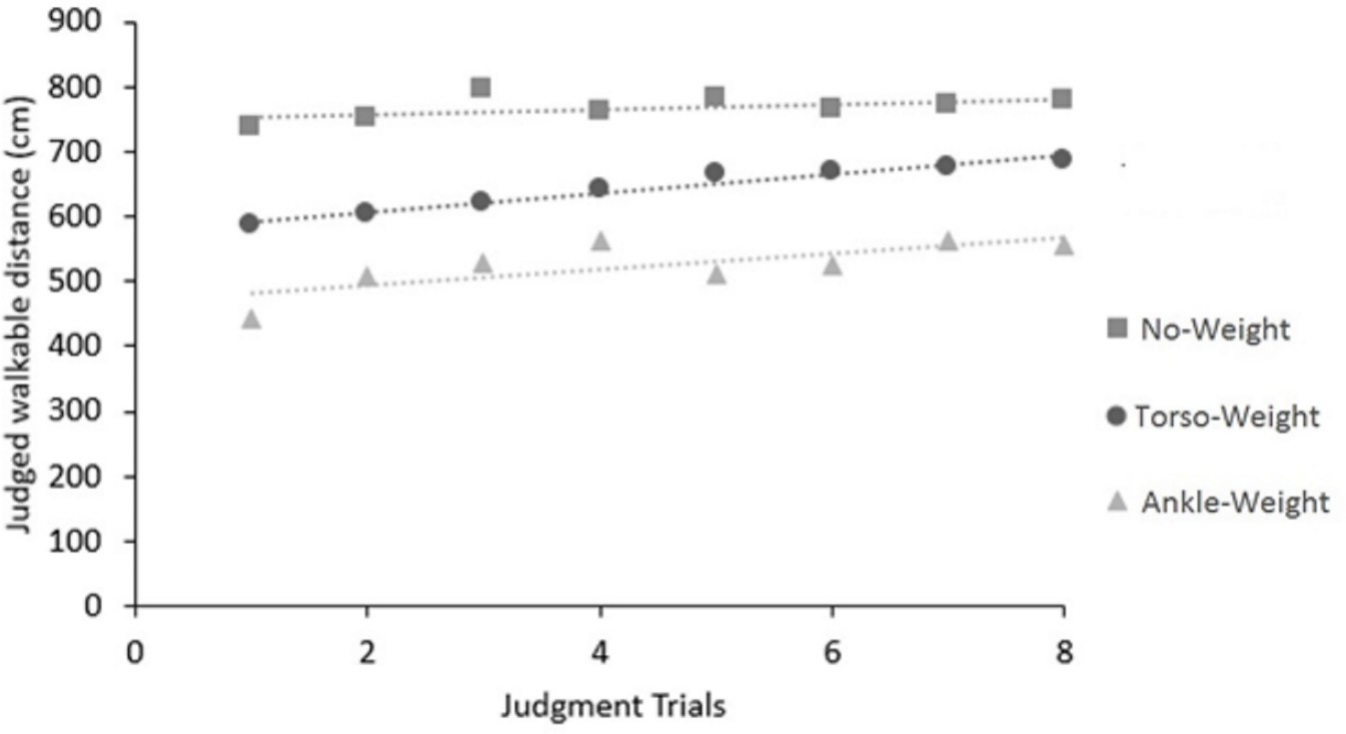
Mean judgments of walkable distance (across participants) as a function of conditions, and judgment trials.

The data are presented in Fig 3. Following Mark et al. [15], we asked whether judged walkable distance changed over the sequence of judgment trials. For each condition, we performed linear regression of judgments across trials. For the No-Weight condition, linear regression yielded a slope of 3.79, which did not differ from 0, *r*^2^ = 0.26, *p* = .19. For the Torso-Weight condition, the slope, 14.76, was greater than 0, *r*^2^ = 0.96, *p* < .001. Similarly, for the Ankle-Weight condition, the slope, 12.02, was greater than 0, *r*^2^ = 0.54, *p* < .05.

### Walking performance

#### Distance

**Fig 4:**
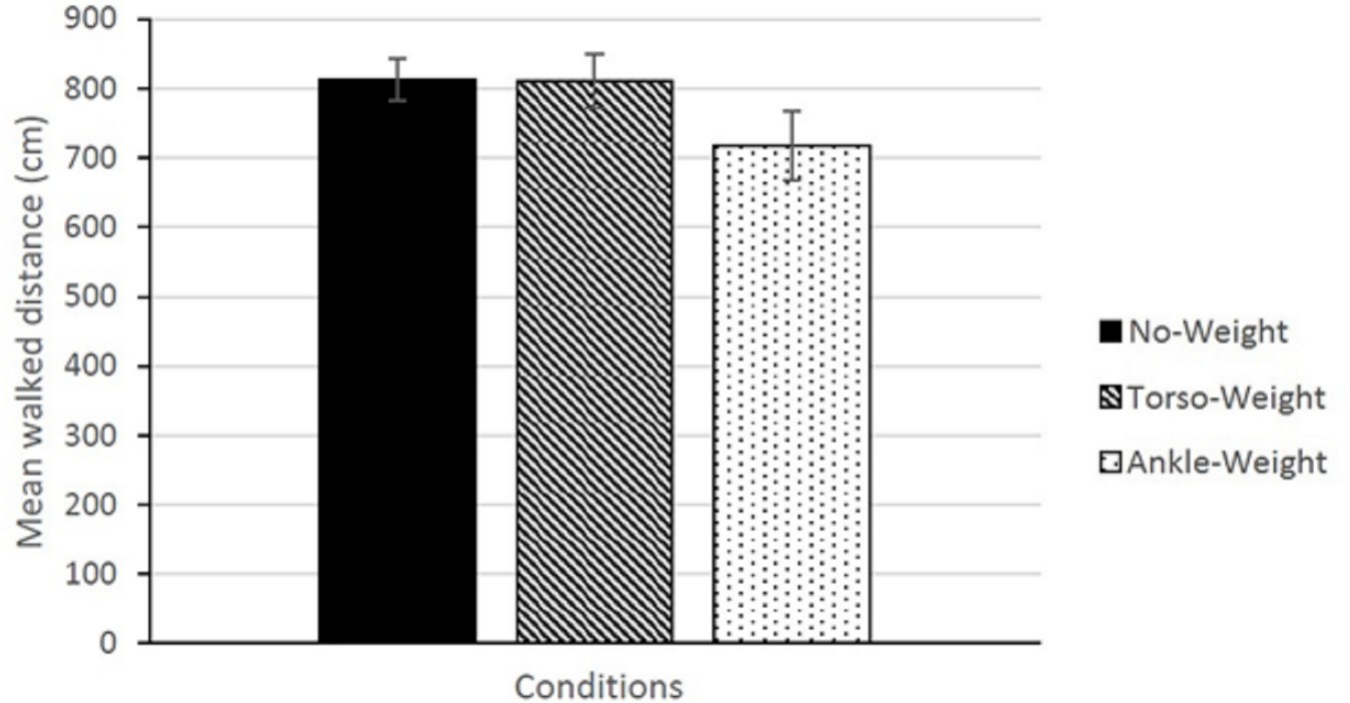
Actual walked distance (mean across participants) as a function of conditions. The error bars represent the standard error of the mean.

The data are summarized in Fig 4. In analyzing the performance trials, we took the mean of the 12 trials for each condition. Using these means, we conducted a 3 × 6 repeated measures ANOVA with factors Conditions (No-Weight, Torso-Weight, Ankle-Weight) and Condition Order (1–6). The main effect of Conditions was significant, *F*(2,16) = 4.64, *p* = .026, partial η^2^ = 0.367. The main effect of Condition Order, and the Conditions × Condition Order interaction were not significant.

#### Speed

**Fig 5:**
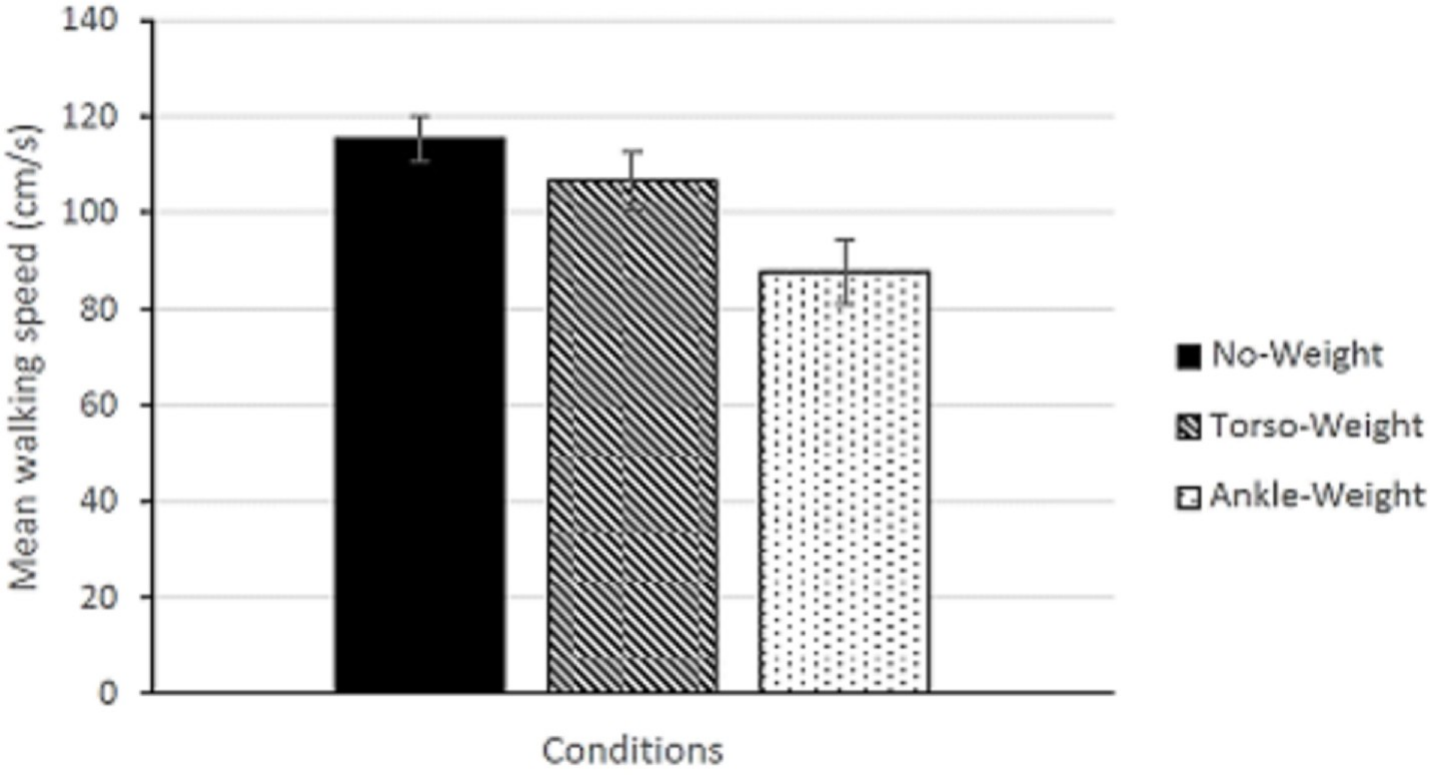
Walking speed (mean across participants) as a function of conditions. The error bars represent the standard error of the mean.

Using data on distance walked and duration, we computed walking speed for each performance trial. These data are summarized in Fig 5. In analyzing walking speed, we took the mean of the 12 trials for each condition. Using these means, we conducted a 3 × 6 repeated measures ANOVA with factors Conditions (No-Weight, Torso-Weight, Ankle-Weight) and Condition Order (1–6). The main effect of Conditions was significant, *F*(2,16) = 17.90, *p* < .001, partial η^2^ = 0.691. The main effect of Condition Order, and the Conditions × Condition Order interaction were not significant.

## Discussion

In Experiment 1, standing participants made judgments about the distance they could walk along a narrow path. In a within-participants design, each participant made a series of judgments while wearing weights on their ankles, on their upper torso, and in a control condition with no added weight. We predicted that added weight would reduce actual walking ability; that is, we predicted that added weight would reduce both the distance that participants could walk within the narrow lane, and the speed at which they walked. We also predicted that effects of the weights would be reflected prospectively in judgments of maximum walkable distance. Finally, following Mark [14–15], we predicted that, in the added weight conditions, initial judgments would be relatively inaccurate, and that accuracy would improve across the series of judgments, despite the absence of walking practice, or judgment feedback. Each of these predictions was confirmed.

## Experiment 2

Motion of a ship at sea tends to alter walking gait, a phenomenon that has been reported anecdotally for thousands of years [11]. Walter et al. [5–6] showed that oscillatory ship motion altered the ability of experienced maritime crewmembers to walk along narrow paths laid out on the open deck, and that these changes were reflected in prospective judgments of walking ability. In these studies, affordances for walking were influenced by dynamic properties of ship motion; principally, the multi-axis, aperiodic oscillations of the ship under the influence of wind and waves. Because it is aperiodic, ship motion is unpredictable, in the sense that individual (i.e., moment-to-moment) motions cannot be predicted. Yet, important characteristics of ship motion are highly predictable. The oscillation frequencies are reliably concentrated in the 0.1–0.4 Hz range [10–11], while oscillation magnitudes are stable (within a certain range) over relatively long periods of time (e.g., hours). This constellation of characteristics is exactly what must be accommodated in getting one’s sea legs. The fact that people get their sea legs, and have been doing so for many millennia, suggests that perceptual-motor systems are robust to the mix of predictability and unpredictability that is oscillatory ship motion.

In Experiment 1 of the present study, we found that participants were prospectively sensitive to changes in walking affordances brought about by changes in the body (added weight) that influenced dynamically the control of walking. In Experiment 2, we asked whether this sensitivity would be preserved when dynamic changes in the body (the wearing of added weights) were coupled with dynamic changes in the environment that also influence walking ability; namely, the aperiodic motion of a ship at sea.

In Experiment 2, our participants were working crewmembers. In consideration of their limited availability, we included only one of the weight conditions that had been used in Experiment 1 (thereby shortening the experimental protocol by approximately one third). In Experiment 1, the largest difference between conditions was between the No-Weight and Ankle-Weight conditions (Fig 2). Accordingly, in Experiment 2 we used these two conditions only. Except as indicated below, in all other respects, the procedure in Experiment 2 was identical to that of Experiment 1.

## Method

### Participants

Our sample comprised 9 individuals (8 men and 1 women), ranging in age from 22 to 62 years (mean = 39.78 years), in height from 1.6 to 2.03 m (mean = 1.75 m) and in weight from 49.9 to 108.8 kg (mean = 81.13 kg), and with 1–37 years (mean = 15.1 years) experience working at sea. Participants were working crew members who volunteered (with the Captain’s permission), taking time off from their regular duties. None of these individuals had participated in our earlier studies [5–6]. The consent process and IRB approval were the same as for Experiment 1. At sea, the number of participants is limited by a number of factors. Testing can be conducted only under appropriate weather conditions; neither calm (such that ship motion would be absent), nor so rough as to prohibit safe walking. For Experiment 2, only one day at sea was suitable for testing. On that day, the number of participants was limited to individuals who choose to volunteer. For these reasons, we computed post-hoc power, which is reported below.

### Setting

The study was conducted during a 5-day cruise aboard the R/V Sally Ride, from San Diego CA to Newport OR. The ship was 86.26 m long with a 15.24 m beam. It displaced 3043 tons, and cruised at 10–12 knots.

### Procedure

The ship departed San Diego CA on June 26 2018, and arrived in Newport OR on June 30 2018. The data were collected on June 29. Data were collected during full daylight, between 9:00 and 17:00.

**Fig 6:**
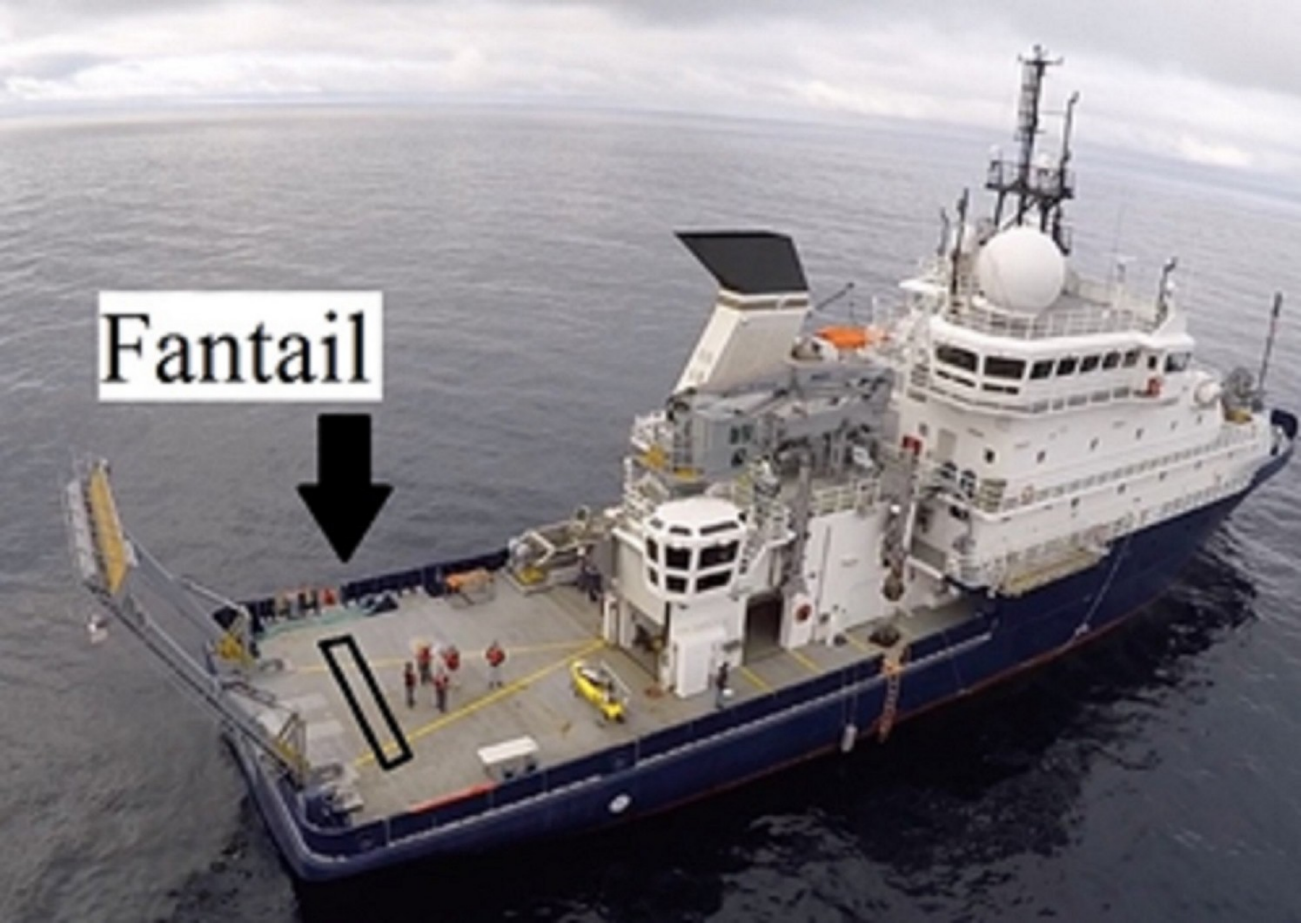
Aerial view of R/V Sally Ride, showing the open rear deck, or fantail. The black rectangle indicates the pathway used in Experiment 2. In the photograph, the pathway is not drawn to scale.

Testing was conducted on the rear deck of the ship (the fantail), which was free from clutter (Fig 6). One pathway (8.9 m long × 0.2 m wide) was created using clearly visible gaffer tape. On the first day at sea, a preliminary assessment suggested that walking was not strongly constrained when the path width was 30 cm. For this reason, in Experiment 2, path width was set at 20 cm. The pathway was parallel to the ship’s short (athwart) axis. Judgment data were collected with the participant standing at one end of the pathway. At this starting location, participants stood with their feet on the taped lines. The purpose was to standardize foot position to reduce variation in the walking distance. We used a within-participants design, in which each individual participated in both conditions.

## Results

During testing, the sea state declined from 7 to 4 on the Beaufort Scale [27]. Anecdotally, during the familiarization phase participants’ gait appeared to be natural and comfortable. By contrast, during the walking performance trials (i.e., after completing judgments), participants often made visible efforts to maximize their performance, such as waving their arms or shortening their stride. That is, in their actual walking performance they appear to have tried to “walk as far as possible”, rather than to “walk comfortably”. We did not exclude these trials from our analysis. Data was deposited in DRUM (Data Repository for University of Minnesota), and can be found online [26].

### Mean judgments

One participant gave the maximum judgment (890 cm) on all trials in both conditions and, for this reason, was deleted from our analysis, leaving a sample size of 9. For each condition, we calculated means for the eight judgments. We conducted a 2 × 2 repeated measures ANOVAs on these values with factors Conditions (No-Weight vs. Ankle-Weight) and Condition Order (No-Weight–Ankle-Weight vs. Ankle-Weight–No-Weight). The ANOVA revealed that the main effect of Conditions was significant, F(1,7) = 8.06, *p* = .025, partial η^2^ = 0.54. The observed power for this effect was 0.684. Judged walkable distance in the No-Weight condition (mean = 535.23 cm, SE = 48.17 cm) was greater than in the Ankle-Weight condition (mean = 429.44 cm, SE = 56.59 cm). In addition, the main effect of Condition Order was significant, F(1,7) = 14.76, *p* = .006, partial η^2^ = 0.68. Across conditions, mean judgments were greater when the Ankle-Weight condition was presented first (mean = 671.07 cm, SE = 73.24 cm) than when the No-Weight condition was presented first (mean = 293.59 cm, SE = 65.51 cm). The Condition × Condition order interaction was not significant.

### Changes across judgment trials

**Fig 7:**
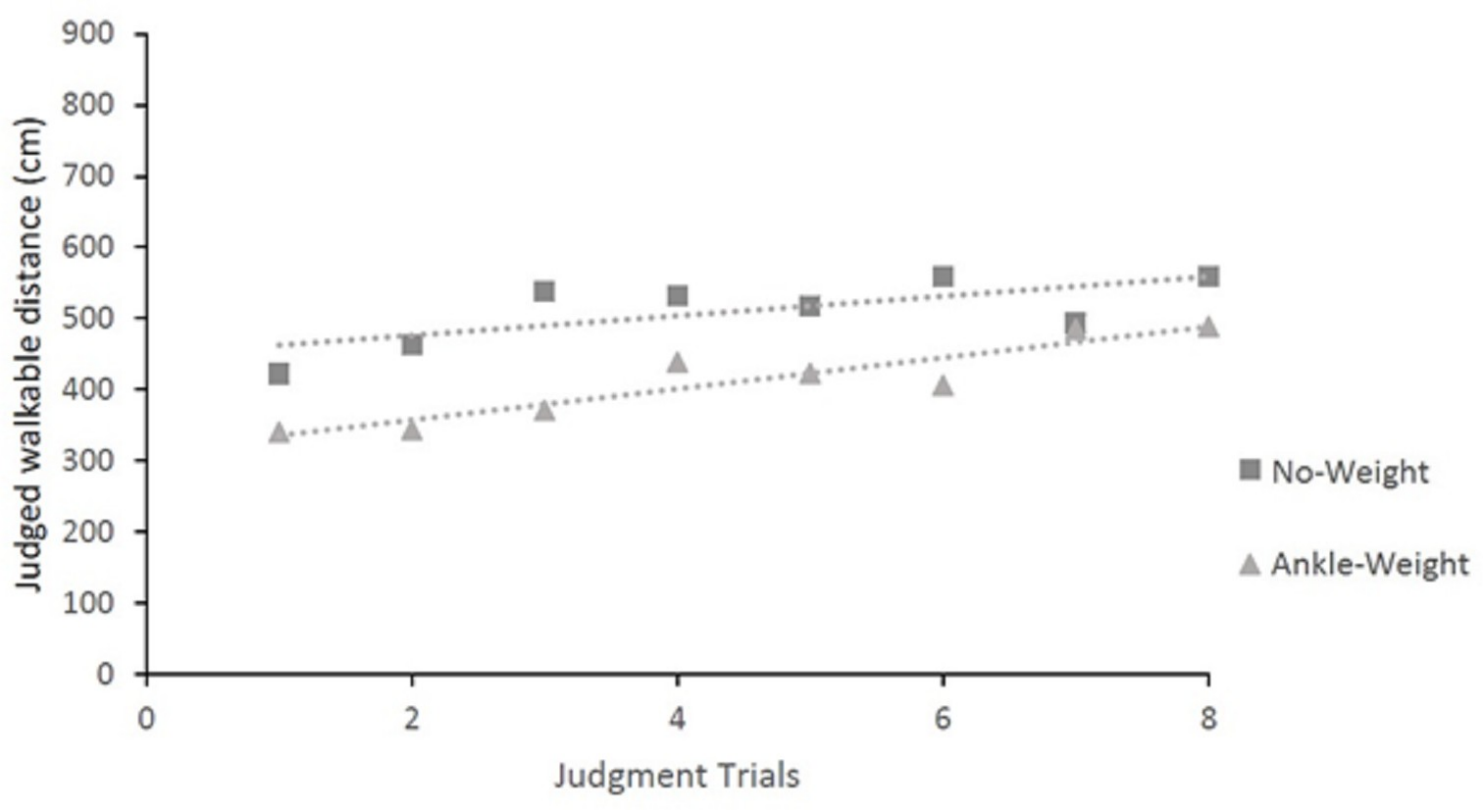
Mean judgments of walkable distance for each condition as a function of judgment trials.

The data are summarized in Fig 7. Following Mark et al. [15], we asked whether judged walkable distance changed over the sequence of judgment trials. For each condition, we used linear regression of judgments across trials. For the No-Weight condition, linear regression yielded a slope of 21.93, which was significantly greater than 0, r^2^ = 0.85, *p* < .001. For the Ankle-Weight condition, the slope, 13.80, did not differ from 0, *r*^2^ = 0.49, *p* > .05.

### Walking performance

#### Distance

We took the mean of the 12 trials for each condition. We conducted a 2 × 2 repeated measures ANOVA with factors Conditions (No-Weight vs. Ankle-Weight) and Condition Order (No-Weight–Ankle-Weight vs. Ankle-Weight–No-Weight). The ANOVA revealed no significant effects.

#### Speed

We took the mean of the 12 trials for each condition. We conducted a 2 × 2 repeated measures ANOVA with factors Conditions (No-Weight vs. Ankle-Weight) and Condition Order (No-Weight–Ankle-Weight vs. Ankle-Weight–No-Weight). The main effect of Condition was significant, F(1,7) = 8.62, *p* = .022, partial η^2^ = 0.552. Speed in the No-Weight condition (mean = 91.86 cm/s, SE = 10.81 cm/s) was greater than in the Ankle-Weight condition (mean = 82.20 cm/s, SE = 9.70 cm/s). There were no other significant effects.

## Discussion

On a ship at sea, we asked experienced maritime crewmembers to judge how far they could walk while remaining within the boundaries of a marked path with no added weight, or while wearing weights at the ankles. Participants judged that they could walk further with no added weight than when wearing the Ankle-Weights. Over the series of eight judgment trials in each condition, judgments changed in the No-Weight condition, more closely reflecting actual walking ability over the course of the eight trials.

Actual distance walked did not differ between the conditions, but participants walked more slowly when wearing the Ankle-Weights than with No-Weight. In the Torso-Weight condition, it might be that the weights, while making walking more effortful (due to the overall increase in mass), also functioned to stabilize the body with respect to ship motion. A related effect was reported by Malek and Wagman [28], who found that wearing a weighted pack on the chest increased the maximum uphill slope on which participants could stand.

The fact that mean judgments were reduced in the Ankle-Weight condition suggests that participants accurately detected the influence of added weight on their walking ability. Walter et al. [5] demonstrated that maritime crewmembers were sensitive to variations in affordances for walking that arose from direction-specific variations in ship motion (walking fore-aft vs. walking athwartship). Walter et al. [6] showed that sensitivity to these direction-specific constraints was itself robust across qualitative changes in ship motion (i.e., in the relative magnitude of roll and pitch). These earlier results demonstrate that participants could detect effects of ship motion on their own walking ability. In Experiment 2 of the present study, judgments varied across conditions despite the fact that, between conditions, there was no systematic difference in the motion characteristics of the ship. Thus, the main effect of conditions in our Experiment 2 suggests that participants differentiated the constraints on walking ability imposed by added weight (which changed across conditions) from those simultaneously imposed by ship motion (which did not). The present result thus extends the findings of Walter et al. [5–6], to the domain of variations in the dynamic properties of the body.

## General discussion

In two experiments, we manipulated properties of the animal and of the environment that would tend to influence the distance that could be walked along a narrow path. In Experiment 1, weights affixed to the body (at the torso, or at the ankles) altered participants’ judgments of the distance that they could walk within a narrow path. Prior to actual walking, judged maximum walkable distance changed over the course of a series of judgment trials when wearing weights, but did not change when no weight was worn. Overall mean judgments differed between the weight conditions, and reflected actual differences in (subsequent) walking ability.

In Experiment 2, we evaluated the “No-Weight” and “Ankle-Weight” conditions on a ship at sea, such that affordances for walking along the narrow path were influenced simultaneously by affixed mass and ship motion (oscillation in pitch). Judged maximum walkable distance again differed between conditions, reflecting actual differences in (subsequent) walking. In the “No-Weight” condition (but not in the “Ankle-Weight” condition), means changed over the course of a series of judgments, suggesting that the task was novel for our participants (experienced maritime crewmembers).

### Adaptive perception of differing constraints

In each experiment, weights added to the body reduced actual walking ability, in terms of distance walked and/or walking speed, consistent with previous research [18–21]. In addition, in each experiment, added weight reduced prospective judgments of walking ability. Previous studies have demonstrated prospective sensitivity to affordances relating to the width of the body (the shoulders, or the midriff) in walking through apertures [29–30]. Our results extended these earlier studies by demonstrating prospective sensitivity to affordances relating to the ability to control lateral placement of the feet in walking.

### Learning across judgments

Mark [14–15] required participants to wear 10 cm blocks on their feet, which increased their actual maximum sitting height. While wearing the blocks, initial judgments of maximum sitting height were relatively inaccurate. However, over the series of judgments, accuracy improved. In the present study, we adapted Mark’s method to judgments about maximum distance that participants could walk along a narrow path. Replicating Mark, in Experiment 1 judgments were stable (across judgment trials) in the absence of added weight, but improved when participants wore added weights at the torso, or at the ankles [31]. That is, participants learned about their changed affordance for walking despite having neither practice walking with the weights, nor feedback about judgment accuracy. Thus, on land (Experiment 1), our results resembled those reported by Mark [14–15], extending their method for studying perception of the affordance for sitting to perception of affordances for walking. In Experiment 2, on a ship at sea, we observed a similar effect when participants did not wear added weight, suggesting that our nautical walking tasks was novel to our participants.

Mark et al. [15] showed that improvement in judgments over a series of judgment trials depended upon the availability of ordinary body sway during judgments. That is, body sway appeared to serve an exploratory function, generating information that was sufficient for perception of affordances for sitting. Several studies have shown that learning of affordances was, in fact, related to quantitative details of postural movement [16–17, 32], in particular the degree of multifractality of sway [33–34]. Taken together with our results, these findings motivate future research that includes measurements of postural activity during judgments of walking ability on land, and at sea.

### Affordance categories?

Some researchers often have suggested that affordances naturally fall into separate categories [35]. Affordances that are influenced by relatively static, or geometric properties of the body, such as leg length [14, 36], and shoulder width [30] are often referred to as ‘body scaled affordances.’ Affordances that are influenced by dynamic properties of the body such force production, energy efficiency [36], or running speed [37] are often referred to as action-scaled affordances. However, this categorization has been empirical rather than theoretical, or *a priori* [38]. In fact, in physical terms *static* and *dynamic* are not mutually exclusive; rather, static properties are a limiting case of dynamics [39]. Of equal importance, affordances can be influenced by either (or both) static and dynamic properties of the environment, such as the width of an aperture [40], or the trajectory of a ball in flight [37]. The formal vacuity of a body-scaled versus action-scaled dichotomy is reflected in experimental research showing that affordances that have been formalized in geometric terms (i.e., relatively static body properties, such as leg length) also are constrained by dynamical properties (i.e., such as metabolic efficiency, muscle strength, and joint flexibility [36, 41–42]. Similarly, properties of the body that are static (in the sense of being relatively persistent), in and of themselves, exert influence over action capabilities through their impact on body movement. The present study offers additional evidence that the categorization is misleading, and may be entirely fictitious [29, 39]. More specifically, the empirical distinction between body-scaled and action-scaled affordances is an artifact, or reification of the *a priori* hypotheses and experimental methods that have been studied, and the experimental methods that have been employed [30, 43].

## Conclusion

In two experiments, weights added to the body at the torso, or at the ankles yielded dynamic consequences for walking (lateral foot placement). Affordances related to the weights were detected prospectively, in the absence of either walking practice, or feedback about the accuracy of judgments. On land, judgments were stable across trials in the baseline condition with no added weight, reflecting participants’ typical walking ability. With added weight, initial judgments underestimated actual walking ability but, over the series of eight judgment trials judgments gradually increased in the direction of accuracy. At sea, initial judgments without added weight were underestimates, but again gradually increased over the series of judgment trials, suggesting that our task was novel even for experienced maritime crewmembers.

The results of the two experiments are consistent with the hypothesis that non-performatory movements, made before participants provided judgments, generated information about how the weights changed walking ability, and that participants’ prospective judgments were informed by this self-generated information [14–15]. In Experiment 2, this was true despite the fact that both judgments and actual walking occurred in the presence of complex, multidimensional oscillation of the ground surface (a ship at sea). Overall, our results suggest the presence of robust, prospective sensitivity to the dynamic influence of added weight on affordances for walking.

## References

[1] Gibson JJ. The ecological approach to visual perception. Boston: Houghton Mifflin; 1979.

[2] Stoffregen TA. Affordances as properties of the animal-environment system. Ecological Psychology, 2003;15: 115–134.

[3] Lee DN, Young DS, McLaughlin CM. A roadside simulation of road crossing for children. Ergonom. 1984;27: 1271–1281.

[4] Plumert JM, Kearney JK, Cremer JF, Recker KM, Strutt J. Changes in children’s perception–action tuning over short time scales: Bicycling across traffic-filled intersections in a virtual environment. J Exp Child Psychol. 2011;108: 322–337. 10.1016/j.jecp.2010.07.005 20728090 PMC2991535

[5] Walter H, Wagman JB, Stergiou N, Erkmen N, Stoffregen TA. Dynamic perception of dynamic affordances: Walking on a ship at sea. Exp Brain Res. 2017;235: 517–524. 10.1007/s00221-016-4810-6 27787584 PMC5297405

[6] Walter HJ, Li R, Wagman JB, Stoffregen TA. Adaptive perception of changes in affordances for walking on a ship at sea. Hum Move Sci. 2019;64: 28–37. 10.1016/j.humov.2019.01.002 30641457

[7] Maki B. Gait changes in older adults: predictors of falls or indicators of fear. J Am Geriatr Soc. 1997;45: 313–320. 10.1111/j.1532-5415.1997.tb00946.x 9063277

[8] Wollensen B, Voelcker-Rehage C. Differences in cognitive-motor interference in older adults while walking and performing a visual-verbal Stroop task. Front Aging Neurosci. 2019;10: 426 10.3389/fnagi.2018.00426 30687077 PMC6333862

[9] Erlandson JM. The archaeology of aquatic adaptions: Paradigms for a new millennium. J Archaeol Res. 2001;9: 287–350.

[10] Wertheim A. Working in a moving environment. Ergonom. 1998;41: 1845–1858. 10.1080/001401398186018 9857842

[11] Stevens SC, Parsons MG. Effects of motion at sea on crew performance: A survey. Marine Technol. 2002;39: 29–47.

[12] Adolph KE, Avolio AM. Walking infants adapt locomotion to changing body dimensions. J Exp Psychol Hum Percep Perf. 2000;26: 1148–1166. 10.1037//0096-1523.26.3.1148 10884014

[13] Regia-Corte T, Wagman JB. Perception of affordances for standing on an inclined surface depends on height of center of mass. Exp Brain Res. 2008;191: 25–35. 10.1007/s00221-008-1492-8 18663440

[14] Mark LS. Eyeheight-scaled information about affordances: A study of sitting and stair climbing. J Exp Psychol Hum Percept Perf. 1987;13: 361–370. 10.1037/0096-1523.13.3.361 2958585

[15] Mark LM, Balliet JA, Craver KD, Douglas SD, Fox T. What an actor must do in order to perceive the affordance for sitting. Ecol Psychol. 1990;2: 325–366.

[16] Stoffregen TA, Yang CM, Bardy BG. Affordance judgments and nonlocomotor body movement. Ecological Psychology, 2005;17: 75–104.

[17] Yu Y, Bardy BG, Stoffregen TA. Influences of head and torso movement before and during affordance perception. J Motor Beh. 2011;43: 45–54. 10.1080/00222895.2010.533213 21218321

[18] Chow DHK, Kwok MLY, Au-Yang ACK, Holmes AD, Cheng JCY, Yao FYD, et al The effect of backpack load on the gait of normal adolescent girls. Ergonom. 2005;48: 642–656. 10.1080/00140130500070921 16087499

[19] Cottalorda JA, Rahmani AB, Diop MA, Gautheron VA, Ebermeyer EA, Belli AA. Influence of school bag carrying on gait kinetics. J Orthoped B. 2003;12: 357–364. 10.1097/01.bpb.0000078270.58527.1f 14530691

[20] Hong Y, Brueggemann G. Changes in gait patterns in 10-year-old boys with increasing loads when walking on a treadmill. Gait Pos. 2000;11: 254–259. 10.1016/s0966-6362(00)00055-2 10802438

[21] Li JX, Hong Y, Robinson PD. The effect of load carriage on movement kinematics and respiratory parameters in children during walking. Europ J Appl Physiol. 2003;90: 35–43. 10.1007/s00421-003-0848-9 12783230

[22] Garciaguirre JS, Adolph KE, Shrout PE. Baby carriage: Infants walking with loads. Child Development, 2007;78: 664–680. 10.1111/j.1467-8624.2007.01020.x 17381796

[23] Lakens D. Calculating and reporting effect sizes to facilitate cumulative science: A practical primer for t-tests and ANOVAs. Front Psychol. 2013;4: 863 10.3389/fpsyg.2013.00863 24324449 PMC3840331

[24] Mayo AM, Wade MG, Stoffregen TA. Postural effects of the horizon on land and at sea. Psychol Sci. 2011;22: 118–124. 10.1177/0956797610392927 21156861

[25] Faul F, Erdfelder E, Lang A-G, Buchner A. G*Power 3: A flexible statistical power analysis program for the social, behavioral, and biomedical sciences. Behav Res Meth. 2007;39: 175–191. 10.3758/BF03193146 17695343

[26] Hannah Walter; Ruixuan Li; Nicolette Peterson; Thomas Stoffregen; Jeffrey Wagman. (2019). APAL "Sensitivity to changes in dynamic affordances for walking on land and at sea" Data Sets. Retrieved from the Data Repository for the University of Minnesota, 10.13020/HFB0-DD13.

[27] Adolph KE. Psychophysical assessment of toddlers’ ability to cope with slopes. J Exp Psychol Hum Percep Perf. 1995;21: 734–750. 10.1037/0096-1523.21.4.734 7643046

[28] Malek EA, Wagman JB. Kinetic potential influences visual and remote haptic perception of affordances for standing on an inclined surface. Quart J Exp Psychol. 2008;61: 1813–1826. 10.1080/17470210701712978 19031153

[29] Franchak JM, Celano EC, Adolph KE. Perception of passage through openings depends on the size of the body in motion. Exp Brain Res. 2012;223: 301–310. 10.1007/s00221-012-3261-y 22990292 PMC3482125

[30] Warren WH, Whang S. Visual guidance of walking through apertures: Body-scaled information for affordances. J Exp Psychol Hum Percept Perf. 1997;13: 371–383. 10.1037//0096-1523.13.3.371 2958586

[31] Ramenzoni VC, Riley MA, Shockley K, Davis T. Carrying the height of the world on your ankles: Encumbering observers reduces estimates of how high an actor can jump. Q J Exp Psychol. 2008;61: 1487–1495. 10.1080/17470210802100073 18609383

[32] Mantel B, Stoffregen TA, Campbell A, Bardy BG. Exploratory movement generates higher-order information that is sufficient for accurate perception of scaled egocentric distance. PLOS ONE, 2015;10(4): e0120025 10.1371/journal.pone.0120025 25856410 PMC4391914

[33] Hajnal A, Clark JD, Doyon JK, Kelty-Stephen DG. Fractality of body movements predicts perception of affordances: Evidence from stand-on-ability judgments about slopes. J Exp Psychol Hum Percep Perf. 2014;44: 836–841. 10.1037/xhp0000510 29809050

[34] Palatinus Z, Kelty-Stephen DG, Kinsella-Shaw J, Carello C, Turvey MT. Haptic perceptual intent in quiet standing affects multifractal scaling of postural fluctuations. J Exp Psychol Hum Percept Perf. 2014;40: 1808–1818. 10.1037/a0037247 24999615

[35] Fajen BR, Matthis JS. Direct perception of action-scaled affordances: The shrinking gap problem. J Exp Psychol Hum Percep Perf. 2011;37: 1442–1457. 10.1037/a0023510 PMC3140555 21500936

[36] Warren WH. Perceiving affordances: Visual guidance of stair climbing. J Exp Psychol Hum Percept Perf. 1984;10: 683–703. 10.1037/0096-1523.10.5.683 6238127

[37] Oudejans RRD, Michaels CF, Bakker FC, Dolne M. The relevance of action in perceiving affordances: Perception of catchableness of fly balls. J Exp Psychol Hum Percept Perf. 1996;22: 879–891. 10.1037//0096-1523.22.4.879 8756956

[38] Pepping G, Li FX. Changing action capabilities and the perception of affordances. J Hum Move Stud. 2000;39: 115–140.

[39] Day BM, Wagman JB, Smith PJK. Perception of maximum stepping and leaping distance: Stepping affordances as a special case of leaping affordances. Acta Psychologica. 2015;158: 26–35. 10.1016/j.actpsy.2015.03.010 25898112

[40] Higuchi T, Cinelli ME, Greig MA, Patla AE. Locomotion through apertures when wider space for locomotion is necessary: Adaptation to artificially altered body states. Exp Brain Res. 2006;175: 50–59. 10.1007/s00221-006-0525-4 16761139

[41] Konczak J, Meeuwsen HJ, Cress ME. Changing affordances in stair climbing: The perception of maximum climbability in young and older adults. J Exp Psychol Hum Percep Perf. 1992;18: 691–697. 10.1037//0096-1523.18.3.691 1500869

[42] Snapp-Childs W, Bingham GP. The affordance of barrier crossing in young children exhibits dynamic, not geometric, similarity. Exp Brain Res. 2009;198: 527–533. 10.1007/s00221-009-1944-9 19626315

[43] Fajen BR. Affordance perception and the visual control of locomotion In: Steinicke F, Visell Y, Campos J, Lecuyer A, editors. Human Walking in Virtual Environments. New York: Springer. 2013 pp. 79–98.

